# Do Changes in Electrical Skin Resistance of Acupuncture Points Reflect Menstrual Pain? A Comparative Study in Healthy Volunteers and Primary Dysmenorrhea Patients

**DOI:** 10.1155/2014/836026

**Published:** 2014-04-27

**Authors:** Yan-Fen She, Liang-Xiao Ma, Cong-Hui Qi, Yan-Xia Wang, Ling Tang, Chun-Hua Li, Hong-Wen Yuan, Yu-Qi Liu, Jia-Shan Song, Jiang Zhu

**Affiliations:** ^1^Beijing University of Chinese Medicine, Beijing 100029, China; ^2^Hebei University of Traditional Chinese Medicine, Shijiazhuang 050200, China; ^3^Hebei General Hospital, Shijiazhuang 050051, China; ^4^Dong Zhi Men Hospital Affiliated to Beijing University of Chinese Medicine, Beijing 100007, China; ^5^Beijing Electric Power Hospital, Capital Medical University, Beijing 100072, China; ^6^School of Traditional Chinese Medicine, Capital Medical University, Beijing 100069, China; ^7^China Academy of Chinese Medical Science, Beijing 100007, China; ^8^The Third Affiliated Hospital of Beijing University of Chinese Medicine, Beijing 100029, China

## Abstract

Electrical skin resistance (ESR) measurements were performed with a four-electrode impedance detector at 10 points bilaterally on the first day of and the third day after menstruation in 48 healthy volunteers and 46 primary dysmenorrhea (PD) patients, to assess whether ESR changes of acupuncture points can reflect menstrual pain or not. The results showed statistical reductions in ESR imbalance ratio between left and right side that were detected at SP8 (Diji) and GB39 (Xuanzhong) (*P* < 0.05), and a statistical increase was detected at SP6 (Sanyinjiao) (*P* = 0.05) on the first day of menstruation compared with those values on the third day after menstruation in dysmenorrhea group. No significant differences were detected at other points within and between two groups (*P* > 0.05). This study showed that the imbalance of ESR at uterine-relevant points in PD patients is not significantly different from those of healthy women on both the 1st day of and the 3rd day after menstruation. The ESR imbalance ratio of certain points can either be lower or higher during menstruation in PD patients. The ESR property of acupuncture points needs to be investigated in further clinical trials with appropriate points, diseases, larger sample sizes, and optimal device.

## 1. Introduction

In classic Chinese acupuncture theory, acupuncture points are not only the sites receiving needling stimulation for treatment, but also the places reflecting the condition of diseases for diagnosis. Electrical skin resistance (ESR) measurements have been used to identify the electrical properties of points and meridians and particularly used for clinical diagnosis. Since 1950s, many studies asserted that points and meridians possess reduced ESR compared to adjacent areas [1–4]. Based on this understanding, it has been assumed that the differences in ESR at acupuncture points may reflect physiologic processes and pathologic conditions in the human body [5, 6]. Numerous devices have been created and widely used for the purpose of locating acupuncture points, diagnosis, and treatment in the clinical practice and research of acupuncture [7–9].

This widely believed explanation of electrical properties of acupuncture points, however, has been questioned yet. Although many studies conducted recently showed that points and meridians had unique electrical properties [10–13], a systematic review found that those studies were generally poor in quality and limited by small sample size and multiple confounders; thus the evidence does not conclusively support the claim that points and meridians are electrically distinguishable [14]. Several studies also showed that the majority of measured points or meridian did not show a changed ESR [15–17], or the phenomenon of low skin resistance does not exist to all acupuncture points [18]; therefore, ESR measurement has been equivocal for acupuncture point localization or diagnostic and therapeutic purposes.

Many technical issues in ESR measurement at points including electrode polarizability, stratum corneum impedance, presence of sweat glands, choice of contact medium, and electrode geometry have been considered gradually by researchers [19]. The controversial results of related studies were mainly caused by ESR measurement devices and measuring procedures. Most of devices are based on a two-electrode method, which may cause significant fluctuation of voltage between the two electrodes due to variable contact impedance between electrodes and tissue. Moreover, variation of pressure on electrodes, angle, or duration of the measurement also can influence the results [10, 12, 13, 16, 17]. However, a four-electrode method can minimize error due to electrode-sample contact resistance, which is serious in two-electrode method [15, 20].

Another important issue in relation to ESR of points is the physiologic and pathologic states of the body. Since acupuncture points are used for both treatment and diagnosis, a comparative investigation between healthy people and patients may reflect the exact electrodermal property of points. Some studies showed correlations between changes in ESR at specific acupuncture points and disease states [21–23]. However, the results of those studies might be questioned by using two-electrode devices.

Chinese acupuncture theory believes that specific acupuncture points, particularly* Yuan*-source points and* Xi*-cleft points, have more significant properties to reflect the physiological and pathological conditions of distant corresponding organ systems [24]. Modern research also showed that certain diagnostic zones on the skin in western medicine had relationship with certain specific points [25]. Our previous study showed that different acupuncture points had different therapeutic effects on primary dysmenorrhea (PD) [26]. Therefore, we propose a hypothesis; that is, ESR imbalance ratio at uterine-relevant points when menstrual pain attacks in PD patients may change significantly more than those in healthy women. In order to assess whether ESR changes of acupuncture points can reflect menstrual pain or not, we conducted this study.

## 2. Materials and Methods

### 2.1. Setting and Participants

Forty-eight (48) healthy volunteers aged 23.67 ± 2.40 yr and 46 patients with primary dysmenorrhea aged 23.76 ± 2.59 yr were recruited on the campus of Beijing University of Chinese Medicine (BUCM, Beijing, China), where the trial was conducted. All volunteers signed an informed consent form before participation. The Medical Ethics Committee of BUCM approved the trial.

Eligible participants in dysmenorrhea group met the following inclusion criteria: (1) the diagnostic criteria of primary dysmenorrhea in the Primary Dysmenorrhea Consensus Guideline [27]; (2) age 15–30 years without history of delivery; (3) normal menstrual cycle (28 ± 7 days); (4) course of dysmenorrhea varying from 6 months to 15 years; (5) experienced menstrual pain scoring more than 40-mm on a 100-mm VAS in continuous three menstrual periods prior to the trial; (6) no oral administration of any analgesic nor acceptance of other therapies in 24 h before the trial; (7) no common cold in one week before the trial and with normal body temperature.

Women with secondary dysmenorrhea caused by endometriosis, uterine myomas, endometrial polyps, pelvic inflammatory disease, and other gynecological problems were excluded. Women with scars on the skin at measured points were excluded.

Participants in healthy group had no history of chronic diseases and were healthy at the time of enrolment. Inclusion criteria of age and duration of menstrual cycle were the same as those in dysmenorrhea group. Considering that some Chinese people may have a basic knowledge of acupuncture, participants with history of acupuncture treatment and knowledge of acupuncture were excluded in both groups.

### 2.2. Electrical Skin Resistance Detecting Device

**Figure 1:**
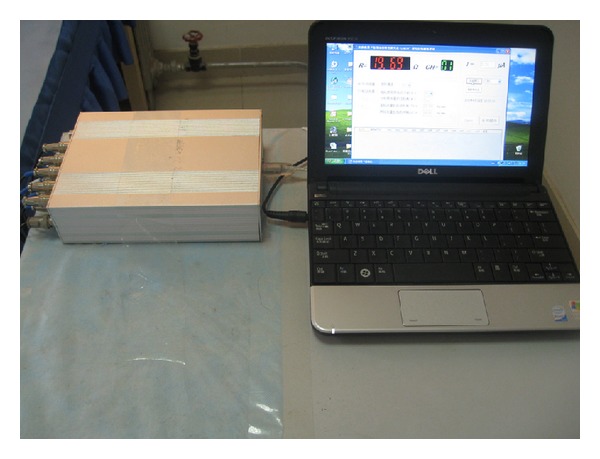
ESR measurement device.

**Figure 2:**
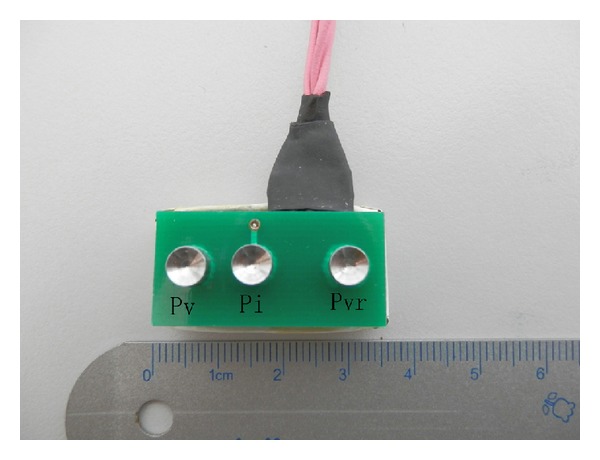
Probe with three electrodes (P_V_, P_I_, and P_VR_).

**Figure 3:**
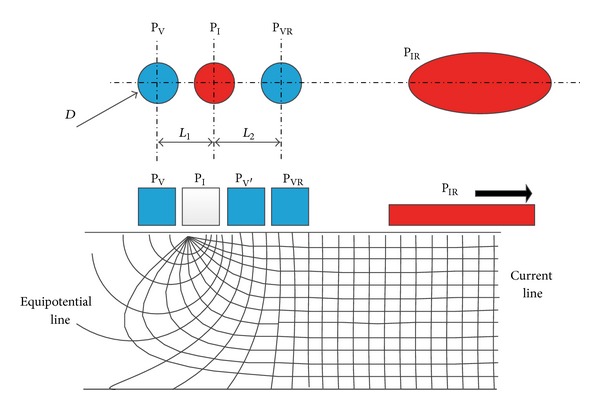
The four electrodes diagram of the ESR detecting device.

**Figure 4:**
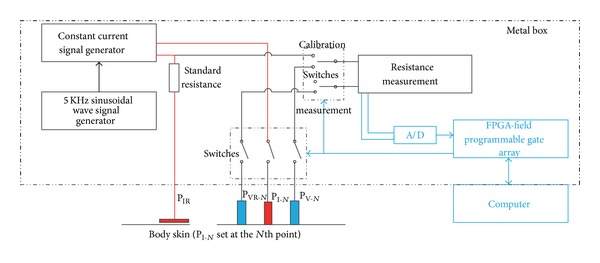
Schematic diagram of the ESR detecting device.

An improved ESR detecting device based on a four-electrode method detector [20] was specially designed and fabricated for this study by School of Physics and School of Electronics Engineering and Computer Science of Peking University (Beijing, China). The new system has been evaluated as a reliable tool for researches on ESR [28]. It consists of two parts, the computer-controlled data obtaining and analyzing system (Figure 1) and 12 metal pipe probes. (Figure 2 shows one probe with three linearly oriented electrodes, which are P_V_, P_I_, and P_VR_ from left to right, respectively.) The four electrodes consist of three small linearly oriented electrodes (P_V_, P_I_, and P_VR_, 5 mm in diameter each, *L*_1_ = 8 mm, *L*_2_ = 12 mm; see Figures 2 and 3) which are sited closely and a reference electrode (P_IR_) which is much larger (50 mm in diameter) and far from the other three electrodes (Figure 3). A common reference electrode (P_IR_) is shared by the 12 linearly oriented electrodes. During the measurement, voltage applied is less than or equal to 300 mV. Signal shape is sinusoidal wave which is generated by the signal generator (see Figure 4). An alternating current (with frequency of 5 KHz and amplitude of 20–30 *μ*A) was delivered to two electrodes (P_I_ and P_IR_) to eliminate tissue polarization, which only can be induced by direct current. Voltage is measured by a voltmeter located between the other two electrodes (P_V_ and P_VR_). The voltage signal was input into a high impedance amplifier and the signal was amplified, filtrated, and sent to a galvanometer and a recorder. The schematic diagram of the device is given in Figure 4; note that for simplicity in the figure only the* N*th probe is shown. The specific arrangement of the electrodes ensures that the device measures only the resistance of the small subcutaneous region lying just below the electrode P_I_. An advantage of such a system is that it can be used to monitor, almost continuously, the ESR variation of up to 12 skin points. Therefore, it can be very useful for studying how the ESR varies under same physiological or pathological conditions.

### 2.3. Detecting Points

According to the theory of Chinese medicine, female reproductive homeostasis is closely related to the organs: “spleen,” “liver,” and “kidney”; therefore, some specific points on spleen, liver, and kidney meridians including three* Xi*-cleft points (SP8, LR6, and KI5), three* Yuan*-source points (SP3, LR3, and KI3), and a nonspecific point (SP10) were chosen as uterine-relevant points. GB39 and an adjacent nonmeridian point located at the midpoint between stomach meridian and gallbladder meridian on the same level of SP6 and GB39 were chosen as uterine-irrelevant points. All acupuncture points were determined according to standards issued by WHO [29]. To ensure consistency of assessment, the above-mentioned points in all participants were located and marked on the skin by the same senior acupuncturist, who has more than 10-year clinical experience in an academic acupuncture clinic, throughout entire study.

### 2.4. Blinding

Participants were blinded to investigation, as those who had history of acupuncture treatment and knew the effects of acupuncture points were excluded. The acupuncturist who put the probes on the points maintained neutral communications with all participants. In the process of trial, the acupuncturist was separated from the ESR measurement technician. Both of them were blinded to measurement allocation.

### 2.5. Outcome Measures

ESR imbalance ratio (absolute differential value of ESR at same points between left and right side/higher ESR at either side) was used as primary outcome measure in this study. The ESR values of each point at left and right side were measured firstly; then we use the following formula to convert the raw data of ESR to imbalance ratios:

(1) $$\begin{array}{c} \text{ESR}\;\text{imbalance}\;\text{ratio}\;=\frac{\left|{\text{ESR}}_{\text{L}}-{\text{ESR}}_{\text{R}}\right|}{\text{Higher}\;\;\text{ESR}\left(\text{L}\;\text{or}\;\text{R}\right)}. \end{array}$$

According to the human body symmetry, significant asymmetry of acupuncture points in terms of ESR, skin temperature, and transcutaneous CO_2_ emission may suggest a pathological condition [30–32]. To measure the difference of those indexes of human bilaterally corresponding points are used as an assistant diagnostic method. ESR asymmetry was expressed by the absolute difference of bilateral points, or the ratio of values on left point and right point with the same name. However, the value of the former one will be influenced by large fluctuation of raw ESR values and cannot be used for comparing different indexes. Our pretest on a 74-year-old healthy male volunteer at 12 points showed a significant large difference of ESR (26.5 Ω–32.67 Ω). It suggested that the ESR difference was not suitable for comparison directly. The later one, ratio of left ESR and right ESR, was not a normalized value; thus, it was also not suitable to reflect the point asymmetry. However, one study showed that balance ratio (difference of measured values between left side and right side/higher value at either side) was a satisfied index reflecting the balance condition of acupuncture points [32]. If the value of the ratio is 0, which may indicate well balance of ESR at one point between left and right sides. If the ratio was 1, it indicated a significant imbalance of ESR. Therefore, we used this imbalance ratio as outcome measure in this study.

### 2.6. Procedure

**Figure 5:**
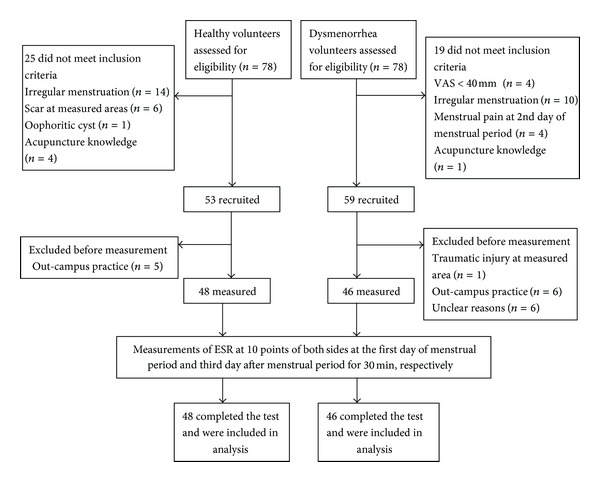
Flowchart of participants through the study.

The procedure of our trial was as follows. (1) Eligible participants were required to contact trial coordinator and arrive at the experimental room on their first day of menstruation (within 24 hr). (2) On arrival, participants acclimatized for 15 min in a standardized room at 22.00 ± 1.42°C and a humidity of 50.48 ± 3.93%. (3) The acupuncturist located all 10 points on both sides and made marks on the surface of skin with ink marker. (4) Glycerine (50%) was used as medium between the electrodes and skin and wiped on 5 points on both sides (LR6-Zhongdu, SP6-Sanyinjiao, GB39-Xuanzhong, KI3-Taixi, and SP3-Taibai, 10 points in total) firstly and then the acupuncturist put 10 probes on those 10 points with current electrodes (P_I_) on the center of measured points and attached by elastic straps with proper pressure. The reference electrode (P_IR_) was bound on the medial side of upper left arm distal to the elbow crease. (5) After 30 min of continuous measurement on first 5 points, 10 probes were moved on another 5 points (SP10-Xuehai, SP8-Diji, nonacupoint, KI5-Shuiquan, and LR3-Taichong) on both sides for another 30 min measurement. (6) On the third day after the period finishes, the same procedure was repeated again at the same time of first measurement. Both the acupuncturist and ESR measurement technician were not allowed to talk with the participant when measurement started.  Figure 5 illustrates the procedure of recruitment and measurement.

### 2.7. Statistical Analysis

Means and standard deviations (SD) and median scores as well as range were determined for ESR difference ratio at the same points between left and right side. The results were analyzed with SAS (version 9.2, SAS Institute, Cary, NC). Median scores were analyzed with the Kruskal-Wallis test to assess differences on the median between two groups on two measurement days and between two days within each group of the study at each point. In this study, we considered *P* ≤ 0.05 to be significant.

## 3. Results

A total of 48 healthy volunteers and 46 patients with PD were recruited into the trial (Figure 5). There were no statistically significant differences when comparing the baseline characteristics between the two groups (Table 1). Thus, the initial condition was comparable in the two groups of the study.

### 3.1. Comparisons of ESR Imbalance Ratio on Two Days within Groups

Statistically significant reductions in ESR imbalance ratio between left and right side were detected at SP8 (Diji) (0.08 ± 0.08 versus 0.12 ± 0.09, *P* < 0.05) and GB39 (Xuanzhong) (0.09 ± 0.07 versus 0.15 ± 0.12, *P* < 0.05), and a statistically significant increase was detected at SP6 (Sanyinjiao) (0.16 ± 0.10 versus 0.13 ± 0.11, *P* = 0.05) on the first day of menstruation compared with those values on the third day after the period finishes in dysmenorrhea group (Table 2). No significant differences were detected at other measured points within groups (*P* > 0.05).

### 3.2. Comparisons of ESR Imbalance Ratio on Two Days between Groups

Between-groups comparisons showed that no significant differences of ESR imbalance ratio were detected at all measured points (*P* > 0.05, Table 2).

### 3.3. Side Effects

No adverse events were reported in any of the two groups during the trial.

## 4. Discussion

According to Chinese acupuncture theory as well as some modern research findings, acupuncture points are believed to be distinguishable in bioelectric properties, pathologic responses, therapeutic effects, and anatomical structures compared to adjacent nonmeridian points [33]. The results of this study partly reflected this widely believed claim of electrodermal property of acupuncture points in acupuncture community. Significant changes of ESR imbalance ratio were only found in three acupuncture points in patients with PD. The ESR imbalance ratios of uterine-relevant points in PD patients did not change significantly more than those in women without PD. We also compared the changes of raw ESR values between PD patients and healthy women; there was still no significant difference. Prior studies also found there were no significant changes of ESR of acupuncture points in healthy subjects [15, 17]. It may suggest that acupuncture points have more sensitive reactions under pathologic states. Recently, acupuncture researchers paid more attention to study the “status” of point, namely, sensitization state and rest state [34, 35]. It is also the core viewpoint of acupuncture point in* Neijing *(the most important classic of TCM). When the human body has disease, acupuncture points on the body surface may be sensitized from a “rest status” with various types of sensitization, such as point heat-sensitization [36–38] and significant plasma extravasation of Evans Blue at points [35]. The result of this study showed another type of acupuncture point sensitization to some extent, namely, electrodermal property. However, since there were no significant different changes in ESR imbalance ratio as well as raw ESR value between PD patients and healthy women, changes in ESR of acupuncture points did not reflect menstrual pain according to this study. Our result was similar to another study, which also showed that there was no significant association between pain intensity and change in ESR of acupuncture meridians [39].

Among 10 measured points, only three specific acupuncture points including crossing point of spleen, liver, and kidney meridians (SP6-Sanyinjiao), influential point of marrow (GB39-Xuanzhong), and* Xi*-cleft point of spleen meridian (SP8-Diji) showed significant changes in ESR imbalance ratio. In acupuncture clinical practice, those three points are commonly used for dysmenorrhea due to their effects of promoting the flowing of qi and blood to relieve pain. The finding in this study also showed that those three points had electrodermal properties in relation to menstruation. To a certain degree, our study has explained why SP6, GB39, and SP8 are more effective for PD in terms of bioelectric properties of points. A study showed that applying moxibustion on sensitized points has achieved better treatment effect [40]. Our previous study focused on the therapeutic effect specificity of SP6 and GB39 and nearby nonmeridian point also showed that the effects of SP6 and GB39 on dysmenorrhea were significantly better than that of nearby nonmeridian point [26]. Therefore, the results of our two studies suggested that needling the acupoints with significant electrodermal property may achieve better treatment effect. Further studies are needed to investigate the changes of ESR at more acupuncture points in relation to different diseases as well as to explore its mechanism, so as to guide acupuncture clinical practice.

Considering the confounding technical factors on ESR measurement, we used a four-electrode method and tried to minimize possible disturbing factors in our measuring procedure. Prior studies that used the old type of this device showed a favorable repeatability [11, 41]. An improved device used in this study with smaller electrodes and computer-control data collection and analysis system enables detecting ESR at 12 points simultaneously at four limbs. The new system's performance is fairly stable even in the presence of various confounding factors such as various pressures on the probe, cleaning the skin with alcohol, and exfoliation [28]. At present, two-electrode method is mainly used in EDSD (electrodermal screening device) in medical community, such as Vegatest made in Germany [2], which is applied to locate acupuncture meridians and assist in diagnosing some diseases [42]. However, the results of diagnosing allergies with the same two-electrode method device were different [42, 43]. Limitations were found in those devices based on two-electrode method in ESR measurement [19]. Compared to a two-electrode method, the four-electrode method has many advantages: (1) it is able to measure electrical impedance approximately 2 mm under the surface of the skin, which is more coincided to the original meaning of acupuncture points [20]; (2) it minimizes error caused by fluctuation in voltage and electrode contact impedance. Therefore, the four-electrode method with some significant modifications has been widely used to measure electrical impedance of biological tissue [28, 44–46]. One prior related study also used a four-electrode method to measure ESR at different meridians [15]. The main differences between the devices used in this study and our study include shape of electrodes and placing order of four electrodes. Four needles were used as electrodes in Ahn's study. Considering that inserting needle into the points may affect the flowing of energy at points resulting in changed ESR, we utilized surface electrodes. Another unique characteristic of our device is the specific arrangement of four skin electrodes (P_V_, P_I_, and P_VR_ in one straight line and P_IR_ was placed far from the other three electrodes; P_V_ was moved to the outer side of P_I_ from its common place; see Figure 3), which ensures that it measures only the resistance of a small subcutaneous region lying just below the current electrode P_I_. Unfortunately, this important modification as well as its unique characters has not been realized by others [14, 47].

Since our study measured the resistance of a region 2 mm under the surface of the skin at acupuncture points [20], marker ink on the skin surface of points could not disturb our results. Moreover, resistance of this region is more suitable to reflect the real electrical property of acupuncture points, which are believed located in a stereo-structure under the surface of the skin [33]. Thus only when the needles were inserted into the skin for a certain depth, better treatment effect could be achieved. Some changes in surface acupuncture meridians of dysmenorrhea patients, such as thermal characteristics, should be investigated in further studies.

Finally, we would like to discuss the limitations of this study. Although it serves as a pilot study, no power analysis was performed which lowers the statistical power of the study. Compared with studying points (uterine-relevant points), a few controls including uterine-irrelevant points and nonmeridian points were observed. Since there are numerous complicated factors involved in the studies on the electrical properties of acupuncture points and meridians such as electrode polarizability, electrode geometry, tissue anisotropy, selection of points for measurement, and pathologic states, future studies present challenges. It needs more efforts and wisdom from both acupuncture clinicians and biomedical engineers.

## 5. Conclusions

This study showed that the imbalance of ESR at uterine-relevant points in PD patients is not significantly different from those of healthy women on both the 1st day of and the 3rd day after menstruation. However, the ESR imbalance ratio of certain acupuncture points can either be lower or higher during menstruation in PD patients. The ESR property of acupuncture points needs to be investigated in further clinical trials with appropriate investigated acupuncture points and diseases, larger sample sizes, and optimal device.

## Figures and Tables

**Table 1:** Baseline characteristics of participants in two groups.

	Group A (healthy group) (*n* = 48)	Group B (dysmenorrhea group) (*n* = 46)	*P* value
Age, years	23.67 ± 2.40	23.76 ± 2.59	0.855
Age of menarche, years	13.29 ± 1.20	13.00 ± 1.09	0.223
Duration of menstrual period, days	5.37 ± 1.04	5.26 ± 1.28	0.638
Duration of menstrual cycle, days	29.60 ± 2.11	28.98 ± 1.58	0.109
Body temperature (first day of menstrual period), °C	36.44 ± 0.22	36.52 ± 0.30	0.137
Body temperature (third day after menstrual period), °C	36.33 ± 0.25	36.46 ± 0.26	0.061

Mean ± SD (standard deviation) is given for each parameter.

*P* values from between-groups comparisons using *t*-test.

Note that there is no statistically significant difference when comparing two groups.

**Table 2:** Comparison of ESR imbalance ratios at same points between left and right side on the first day of menstrual period and third day after menstrual period in two groups.

Points	Group A-1 (healthy group) (*n* = 48)	Group A-3 (healthy group) (*n* = 48)	*P* ^a^ value	Group B-1 (dysmenorrhea group) (*n* = 46)	Group B-3 (dysmenorrhea group) (*n* = 46)	*P* ^b^ value	*P* ^ab-1^ value	*P* ^ab-3^ value
SP10 (Xuehai)								
Mean ± SD	0.15 ± 0.10	0.14 ± 0.09		0.17 ± 0.11	0.13 ± 0.10			
Median	0.15	0.13	0.70	0.14	0.13	0.07	0.31	0.39
Range	0.06–0.20	0.08–0.20		0.10–0.26	0.05–0.18			
SP8 (Diji)								
Mean ± SD	0.09 ± 0.06	0.10 ± 0.06		0.08 ± 0.08	0.12 ± 0.09			
Median	0.07	0.10	0.23	0.05	0.10	0.04	0.40	0.67
Range	0.04–0.13	0.05–0.14		0.03–0.13	0.07–0.17			
LR6 (Zhongdu)								
Mean ± SD	0.13 ± 0.11	0.14 ± 0.08		0.13 ± 0.09	0.12 ± 0.09			
Median	0.09	0.14	0.41	0.11	0.10	0.73	0.86	0.19
Range	0.05–0.20	0.07–0.18		0.06–0.18	0.06–0.16			
SP6 (Sanyinjiao)								
Mean ± SD	0.12 ± 0.10	0.12 ± 0.09		0.16 ± 0.10	0.13 ± 0.11			
Median	0.10	0.09	0.97	0.15	0.10	0.05	0.12	1.00
Range	0.04–0.20	0.04–0.19		0.10–0.22	0.04–0.17			
GB39 (Xuanzhong)								
Mean ± SD	0.10 ± 0.08	0.11 ± 0.08		0.09 ± 0.07	0.15 ± 0.12			
Median	0.10	0.10	0.57	0.08	0.10	0.04	0.55	0.29
Range	0.05–0.15	0.05–0.17		0.05–0.12	0.05–0.22			
Nonacupoint								
Mean ± SD	0.12 ± 0.10	0.12 ± 0.10		0.12 ± 0.09	0.11 ± 0.08			
Median	0.09	0.09	0.87	0.11	0.10	0.48	0.54	0.92
Range	0.05–0.16	0.05–0.15		0.05–0.16	0.04–0.16			
KI5 (Shuiquan)								
Mean ± SD	0.12 ± 0.10	0.12 ± 0.08		0.10 ± 0.10	0.12 ± 0.09			
Median	0.08	0.10	0.99	0.08	0.10	0.23	0.58	0.57
Range	0.04–0.16	0.05–0.14		0.04–0.14	0.06–0.16			
KI3 (Taixi)								
Mean ± SD	0.12 ± 0.11	0.12 ± 0.08		0.13 ± 0.10	0.11 ± 0.08			
Median	0.09	0.10	0.67	0.10	0.10	0.80	0.53	0.81
Range	0.03–0.17	0.05–0.17		0.05–0.20	0.06–0.16			
SP3 (Taibai)								
Mean ± SD	0.17 ± 0.13	0.19 ± 0.16		0.17 ± 0.15	0.20 ± 0.17			
Median	0.14	0.13	0.84	0.12	0.15	0.43	0.72	0.80
Range	0.08–0.23	0.07–0.30		0.07–0.21	0.09–0.23			
LR3 (Taichong)								
Mean ± SD	0.12 ± 0.09	0.10 ± 0.06		0.10 ± 0.06	0.12 ± 0.08			
Median	0.09	0.09	0.41	0.08	0.09	0.17	0.31	0.23
Range	0.05–0.17	0.04–0.14		0.04–0.13	0.06–0.17			

ESR imbalance ratios at all points on the first day of menstrual period in healthy volunteer group are given in column of Group A-1, while those on the third day after menstrual period are given in column of Group A-3. Corresponding values of dysmenorrhea group on those two days are given in column of Groups B-1 and B-3, respectively.

*P* ^a^ values are from within-group comparisons of group A on two days using Kruskal-Wallis Test.

*P* ^b^ values are from within-group comparisons of group B on two days using Kruskal-Wallis Test.

*P* ^ab-1^ values are from between-group comparisons of groups A and B on the first day of menstrual period using Kruskal-Wallis test.

*P* ^ab-3^ values are from between-group comparisons of groups A and B on the third day after menstrual period using Kruskal-Wallis test.
